# Recent advances in understanding circular RNAs

**DOI:** 10.12688/f1000research.25060.1

**Published:** 2020-06-29

**Authors:** Constanze Ebermann, Theodor Schnarr, Sabine Müller

**Affiliations:** 1 Institute for Biochemistry, University Greifswald, Greifswald, Germany

**Keywords:** Biogenesis, cancer, circRNA, disease, splicing

## Abstract

Exonic circular RNAs (circRNAs) have been discovered in all kingdoms of life. In many cases, the details of circRNA function and their involvement in cellular processes and diseases are not yet fully understood. However, the past few years have seen significant developments in bioinformatics and in experimental protocols that advance the ongoing research in this still-emerging field. Sophisticated methods for circRNA generation in vitro and in vivo have been developed, allowing model studies into circRNA function and application. We here review the ongoing circRNA research, giving special attention to recent progress in the field.

## Introduction

Exonic circular RNAs (circRNAs) constitute a large class of regulatory non-coding endogenous RNAs with variable composition. Over the past few years, research into their biogenesis and biological function has exploded. First discovered in viroids, where they appear as circular genomes ^1^, circRNAs have been shown to exist in all kingdoms of life, with thousands of circRNAs identified across species from archaea to humans ^2, 3^. For decades, circRNAs were considered to be extremely rare in nature and, in particular in eukaryotes, they were seen as minor RNA structural variants attributed to transcriptional noise ^4^. Owing to progress in analytical techniques and the development of specific methodologies for the discovery and identification of circRNAs (recently reviewed in 5), this picture has dramatically changed over the past several years. It became obvious that circRNAs are abundant, evolutionarily conserved, and stable species in all eukaryotes studied today, although some eukaryotes like *Saccharomyces cerevisiae* have only very few circRNAs because of their few multi-intronic genes. The biogenesis and full functional repertoire of circRNAs have not yet been fully elucidated. Here we will review recent progress in circRNA research, focussing on new data regarding their biogenesis, cellular function, and involvement in diseases. We will extend our view to strategies for controlled generation of circRNAs *in vivo* and *in vitro* and discuss putative applications. We do not include the development of tools and biochemical methods for the accurate identification and characterization of circRNAs, since this, as mentioned above, has been extensively reviewed very recently ^5^.

## Biogenesis of circRNAs

**Figure 1.**
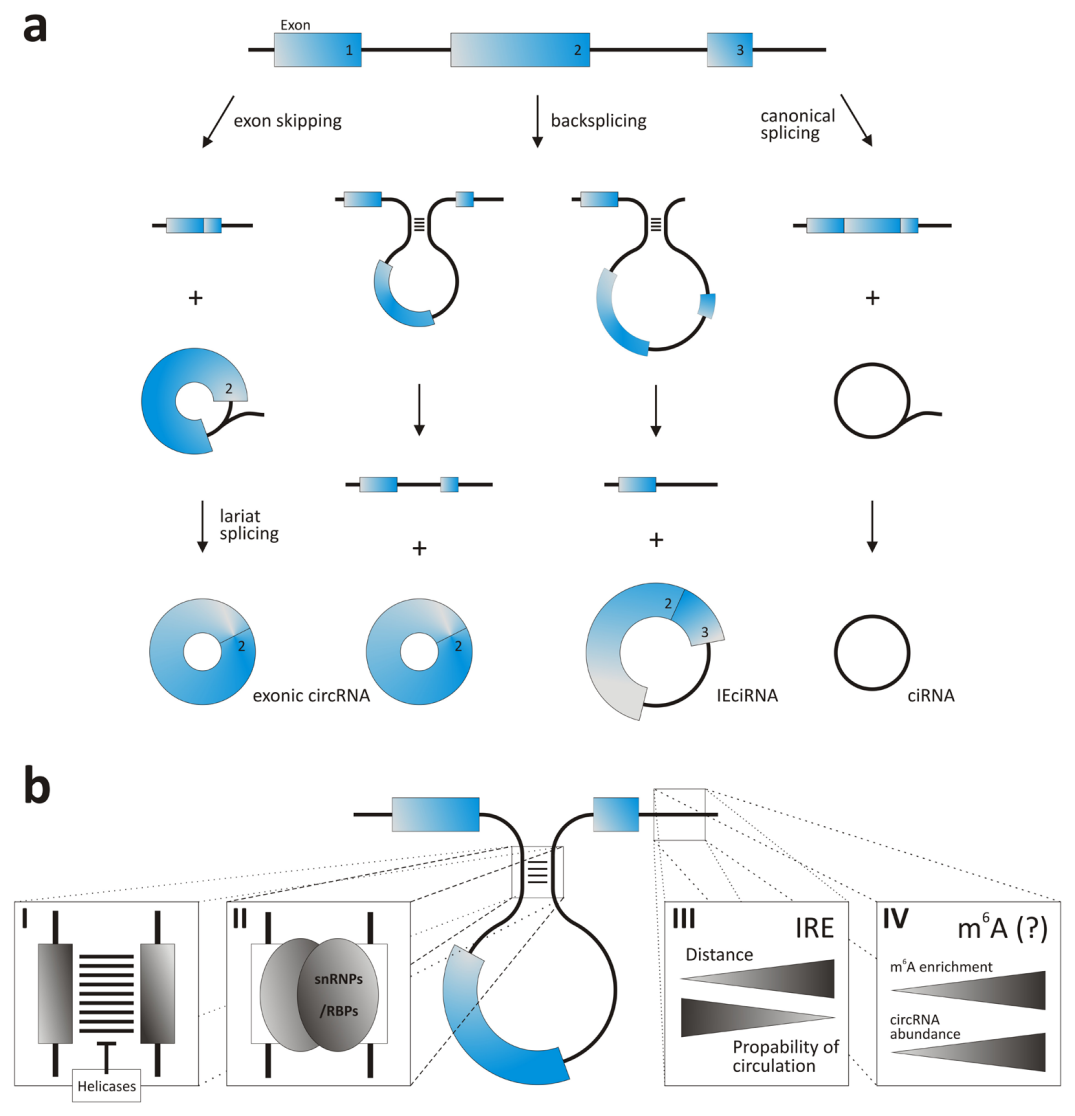
Biogenesis of circRNAs. ( **a**) Modes of circRNA formation. ( **b**) Factors supporting backsplicing: inverted repeat sequences (I), binding sites for RBPs of RNPs (II), IRE distance (III), and m ^6^A-enriched sites (IV). For further explanation, see main text. ciRNA, circular RNA containing sequences from introns; circRNA, circular RNA; IEciRNA, circRNA containing sequences from exons with introns retained between the exons; IRE, inverted repeat; RBP, RNA-binding protein; snRNP, small nuclear ribonucleoprotein.

Most circRNAs are expressed from known protein-coding genes and are composed of single or multiple exons ^3^. They are produced by backsplicing, a process that occurs in a reversed orientation as compared with canonical splicing. Hence, instead of joining an upstream 5'-splice site with a downstream 3'-splice site in a sequential order to produce a linear RNA, a downstream 5'-splice site is linked to an upstream 3'-splice site to yield a circRNA ( Figure 1a) ^6– 9^. Still, the formation of circRNA was shown to be dependent on the canonical splicing machinery, making backsplicing a process that competes with canonical splicing ^10, 11^. In addition to exonic circRNAs, circular RNAs containing sequences from introns (ciRNA) and circRNAs containing sequences from exons with introns retained between the exons (exon-intron circRNA or short IEciRNA) have been found. ciRNAs presumably result from intron lariats that escaped de-branching during canonical splicing and do not belong to circRNAs. They reside in the nucleus, where they may control the transcription of their parental genes ^12, 13^. However, previous work also suggests that some ciRNAs are stable in the cytoplasm ^14, 15^. Exonic circRNAs localize to the cytoplasm, where they are exported from the nucleus in a length-dependent manner ^16^. In general, all exons found in linear transcripts may appear in circRNAs. However, it is also possible that circRNAs contain exons which do not appear in linearly spliced transcripts ^17^.

Successful backsplicing requires the splice sites to be brought into proximity ( Figure 1b). This often is supported by inverted repeats (IRE), especially Alu elements, flanking the exons to be circularized and allowing multiple circRNAs to be produced from a single gene ^8, 18, 19^. In addition to IRE, interaction of the precursor mRNA with ribonucleoproteins (RNPs) or proteins was found to support circRNA formation ^11, 20– 23^. Other RNA-binding proteins (RBPs) that support circularization are, for example, the heterogeneous nuclear RNP L (HNRNPL) ^24^, double-stranded RNA-binding domain containing immune factors NF90/NF110 ^25^, or DHX9, an abundant nuclear RNA helicase ^26^. Moreover, circRNA biogenesis underlies the combinatorial control of splice factors ^21^ and can also be suppressed by helicases ^6, 27^. Pre-mRNA structure plays an important role, as flanking sequences (e.g. IRE or RNP-binding sites) or the distance between splice sites is most important ^28^. Furthermore, N6-methylation of adenosine can promote circRNA biogenesis, as it was recently shown that m ^6^A-enriched sites guide backsplicing in male germ cells ^29^. CircRNA levels are also modulated by the levels of core spliceosome components ^30^, and it was suggested that the same spliceosome can assemble across an exon and that it either remodels to span an intron for canonical linear splicing or catalyses backsplicing to generate circRNA ^31^.

Backsplicing is less efficient than linear splicing ^32^, and, typically, circRNAs are produced at a lower level than their linear counterparts. Yet circRNAs may be the more abundant isoform in specific cells and tissues ^33, 34^, which may be attributed to their higher stability. Owing to the covalently closed ring structure, circRNAs are resistant to degradation by exonucleases, thus undergoing slower turnover. The higher stability implies that possible functions of circRNA may be associated with their longer lifespan. Nevertheless, there is evidence of circRNA turnover, as it was shown that upon poly(I:C) stimulation or viral infection, circRNAs are globally degraded by RNase L, a process required for PKR activation in early cellular innate immune responses ^35^. Furthermore, m ^6^A-containing circRNAs, when bound to the m ^6^A reader protein YTHDF2, become rapidly degraded by the RNase P/MRP complex ^36^. circRNA degradation is also mediated via a structure-related RNA decay pathway that is independent of specific single-stranded sequences but recognizes double-stranded structures in the 3' UTR of mRNAs, as well as highly structured circRNAs ^37^.

## Biological functions of circRNAs

To date, biological function has been investigated for only a minor fraction of circRNAs. Many of those have been proposed to act as miRNA sponges ^38– 42^ or protein sponges ^11, 43^. In addition, circRNAs may enhance protein function ^13, 33^, assist protein target interaction ^44– 46^, or recruit proteins to specific locations ^47^. An early example for a potential miRNA sponge is circRNA ciRS-7, also known as CDR1as, comprising over 70 binding sites for miR-7 ^40^. However, this function is still controversially discussed, in particular when looking at stoichiometric ratios of the target sequences to the number of binding sites in the circRNA ^48, 49^. Furthermore, analysis of 7,000 human circRNAs revealed that most of them are not enriched in miRNA-binding sites ^3^.

Some circRNAs possess binding sites for specific proteins, which upon binding lose interaction with other targets ^50^. In a similar manner, circRNAs have been described to function as protein scaffolds, assisting the assembly of protein complexes ^43, 44, 46, 51^. For example, circFoxo3 was shown to inhibit the progression of the cell cycle by formation of a ternary complex with CDK2 and p21, thereby acting as a tumor suppressor ^46^, or to specifically recruit the ubiquitinylation system, thus triggering degradation of mutated p53 by the proteasome complex ^44^. CircRNA can also regulate the subcellular localization of specific proteins, as shown for circ-Amotl1 binding to Stat3, AKT1, and PDK1 ^45, 52^. Because backsplicing competes with canonical splicing, the formation of circRNAs is also considered to be a mode of regulating the expression of a specific gene. The protein Muscleblind (MBL) binds to the flanking introns of circMBL derived from the muscleblind gene by backsplicing. As a result, MBL levels are modulated, which in turn strongly affects circMBL biosynthesis ^43^.

There has been some indication that cells can differentiate between endogenous and exogenous circRNA. Exogenously introduced circRNA was shown to have a stimulating effect on the immune system because it is recognized by the pattern recognition receptor retinoic acid inducible gene I (RIG-I), thereby eliciting a strong immune response. Apparently, this applies only to unmodified circRNA because m ^6^A-modified circRNA was shown to inhibit innate immunity ^53^. Endogenous circRNA, on the contrary, did not show such an effect. Based on this observation, it was proposed that endogenous circRNA is recognized as self, owing to the identity of its flanking introns that led to circularization ^54^. More recent findings, however, are contradictory, as they suggest that unmodified exogenous circRNA is able to bypass cellular RNA sensors and thus does not induce an immune response in RIG-I and Toll-like receptor (TLR) competent cells and in mice ^55^. Endogenous circRNAs can collectively bind and suppress activation of the double-stranded RNA (dsRNA)-activated protein kinase PKR, thereby controlling innate immune responses ^56^. As already mentioned above, double-stranded RNA-binding domain-containing immune factors NF90/NF110 are key regulators in circRNA biogenesis, pointing to the role of circRNAs in immunity. Upon viral infection, circRNA expression is decreased, and NF90/NF110 released from circRNP complexes bind to viral mRNAs as part of their functions in antiviral immune response ^25^.

Interestingly, some circRNAs containing internal ribosome entry site (IRES) elements and AUG sites may be translated into unique peptides under specific conditions, in particular upon cellular stress ^52, 57– 61^, although the functional relevance of the majority of circRNA-derived peptides is not yet known. Earlier studies had suggested that circRNAs might be translated without the existence of an IRES sequence, following the so-called rolling circle translation mechanism ^62^. In more recent studies, however, translation of circRNAs was shown to be dependent on the presence of different IRESs (either viral IRES sequences ^36, 37^ or m ^6^A ^28^). Yet it should be noted that even though several studies have reported cases of circRNA translation, others have completely failed to find evidence ^63^.

Taken together, circRNAs appear to play a regulatory role in different levels of gene expression, which also explains their association with diverse diseases, pathological conditions, and expression patterns specific for certain cell types and tissues.

## CircRNAs in diseases

CircRNAs have been associated with the initiation and progression of several diseases, including cancer, neurodegenerative diseases, cardiovascular diseases, and diabetes ^51, 64– 69^, and thus have also been considered as biomarkers for disease prognostics and diagnostics and as targets or tools for disease treatment ^70– 72^. There is also indication of circRNAs accumulating with aging ^73– 75^. Work in the field is currently centered around screening for and identifying disease-associated circRNAs, whereas the underlying mechanisms of action remain mostly unknown. In particular, the involvement of circRNAs in cancer development and progression is obvious, as numerous circRNAs have been discovered to upregulate or downregulate gene expression in cancer tissues and promote cancer cell reproduction ^35, 66, 76– 83^. Over the past two years, numerous circRNAs have been shown to affect cell proliferation, invasion, migration, and apoptosis and have been suggested to act as therapeutic targets or biomarkers for diagnosis and prognosis in various types of cancers ^84– 90^. There have been indications of circRNAs occurring in the tumor microenvironment ^91^ and in exosomes ^92^, with their role in cell-to-cell communication and spreading of pathological processes continuing to be unveiled ^92– 94^. Recent results have shown that circRNA-loaded exosomes promote cell proliferation and invasion in colorectal ^95^ and prostate ^96^ cancer. It has been suggested that the effect of extracellular circRNAs can be reversed by the addition of siRNAs targeting those circRNAs, hence making it a promising therapeutic strategy ^96, 97^.

Standing out in the majority of research of "more classical" diseases is the increasing knowledge of the roles of circRNAs in aging, where age-related changes in splicing, and thus in the level of lncRNAs and circRNAs, are discussed ^98^. Furthermore, it has been found that the expression of circRNAs can be sensitive to different types of pollution (organic, heavy metal, and others) and therefore might be used as a biomarker or prevention/treatment target for pollution-induced diseases ^99^.

## Strategies for controlled generation of circRNAs *in vitro* and *in vivo*

**Figure 2.**
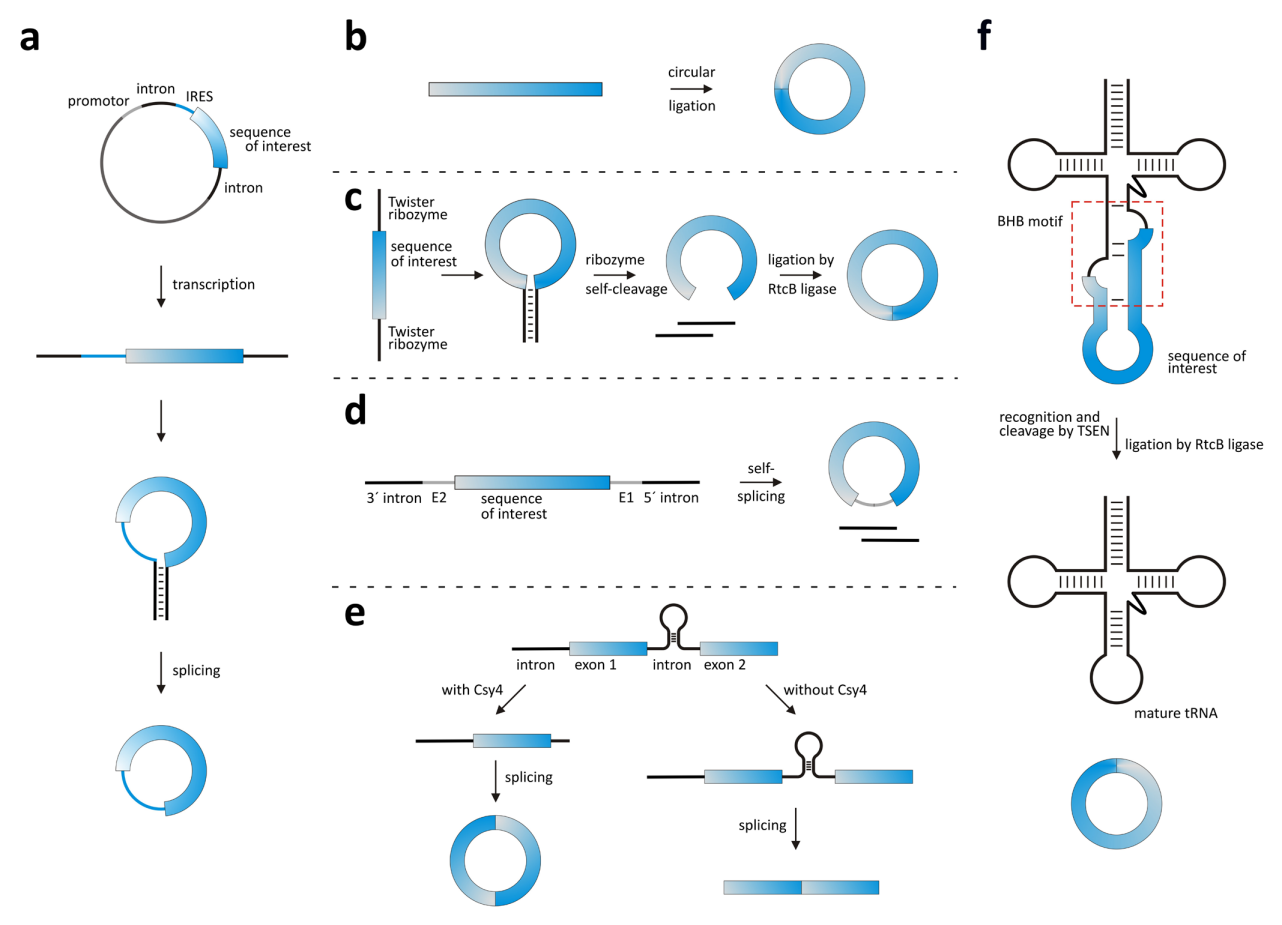
Strategies for circRNA generation *in vitro* and *in vivo*. ( **a**) Overexpression vector, ( **b**) chemical or enzymatic ligation, ( **c**) circularization via Tornado system, ( **d**) permuted intron exon (PIE) strategy, ( **e**) induction of backsplicing by Csy4, and ( **f**) generation of circRNA using tRNA splicing mechanism. For further explanation, see main text. BHB, bulge-helix-bulge; IRES, internal ribosome entry site; TSEN, tRNA splicing endonuclease complex.

Several methods for controlled generation of circRNAs based on chemical or enzymatic/ribozymatic strategies have been investigated ^100, 101^. Circularization can be performed either *in vivo* or *in vitro*. For direct production of circRNA in cell culture, usually the sequence of interest is cloned into an artificial exon that is flanked by complementary intronic repeats. The plasmid is then transfected into cells, transcription is induced, and the cellular splicing machinery generates the desired circRNA ^19, 102^ ( Figure 2a). Alternatively, the sequence of interest can be cloned in between a permuted self-splicing intron (permuted intron exon [PIE] strategy, see below), such that circularization occurs by the inherent ribozyme activity of the intron ^103, 104^. An expression vector comprising such self-splicing introns is also suited for circularization *in vitro* by linearization of the plasmid and subsequent *in vitro* transcription of the linearized template. The formed transcript undergoes circularization by its self-splicing activity ^103^.

Chemical ligation methods can be applied only for *in vitro* circularization. Linear RNA obtained from chemical synthesis or *in vitro* transcription and phosphorylated at the 3'- or 5'- terminus can be intramolecularly ligated with the help of condensing agents ^100^. In addition, enzymatic ligation with DNA or RNA ligases is an option ( Figure 2b) ^100, 101^.

Recently, a seminal approach for circRNA production *in vivo*, called Tornado (twister optimized RNA for durable overexpression), was introduced ( Figure 2c) ^105^. The twister ribozyme is employed in a combined approach with the cellular RtcB ligase. The ribozymes flanking the sequence to be circularized generate by cutting themselves off the 5'-terminal OH and 3'-terminal phosphate required by the cellular RtcB ligase to perform the following ligation step.

Already known for a while and newly moved into focus by recent studies is the PIE strategy, which uses a group I self-splicing intron (also a ribozyme) for the production of a circRNA either *in vitro* or *in vivo* ^106^. The two intron halves (5'- and 3'- intron) flanking the exon are arranged in a permuted manner, such that during splicing a circularized exon and two linear intron halves are formed ( Figure 2d). The PIE strategy was successfully applied for RNA sequences up to five kilobases, and a PIE-produced circRNA carrying an IRES sequence was shown to be successfully translated in cells ^52, 104, 107^.

Another possibility to selectively circularize RNA sequences is utilizing the tRNA splicing machinery ^108, 109^. A tRNA precursor is specifically recognized by the tRNA splicing endonuclease complex (TSEN) based on a bulge-helix-bulge (BHB) motif, then cleaved and ligated by a ligase, yielding the mature tRNA and a circularized intron ( Figure 2f). A desired sequence can be introduced in such a construct between the two intron halves to become circularized upon tRNA splicing. Still another method exploiting the cell’s own splicing machinery for circularization is the system based on RNA cleavage by the CRISPR endonuclease Csy4 ^110^. Csy4 recognizes a 16-nucleotide hairpin in RNA and specifically cleaves off the RNA downstream of that hairpin region. The protein is utilized for RNA circularization to cleave a site in a defined intron, thereby removing a competing downstream splice site, which otherwise would interfere with backsplicing, and thus inducing formation of the desired circRNA ( Figure 2e) ^110^.

## Application of circRNAs

After research in the field of circRNA was dominated by their identification and studies into biogenesis and function, reports on the application of circRNAs have started to emerge more recently. Because of their stability and association with diseases, endogenous circRNAs are potential candidates as biomarkers or therapeutic targets ^111– 113^. Likewise, exogenous circRNAs can be introduced into cells to fulfil a defined function. Several feasible concepts for the therapeutic application of circRNAs have already been discussed and to some extent successfully implemented. An obvious possibility for the application of circRNAs is the development of designed miRNA sponges. An artificial circRNA molecule comprising multiple binding sites for miRNA-122, which plays an essential role in the life cycle of the hepatitis C virus, was successfully used to inhibit the synthesis of viral proteins in the host cell ^114^. In a similar way, the activity of specific proteins in the cellular context was controlled by circularized aptamers ^105^. Moreover, circular aptamers have shown great potential as intracellularly expressed biosensors for defined metabolites ^103, 105^.

Because some circRNAs play a role in alternative splicing and transcription, it is feasible to use them for the regulation of those processes within the cell, thereby driving gene expression in the direction of specific transcription and splicing products. In addition, circularization of RNA opens up the opportunity to apply RNA therapeutics that are administered as a linear construct until now (for example, mRNA vaccines) in a circular form, thereby significantly increasing their stability. If the circRNA additionally possesses an IRES sequence, translation of that RNA is possible, whereby therapeutic proteins may be expressed directly in target cells. Because of results suggesting that circRNAs can activate the immune system via the RIG-I pathway, it is also feasible to employ exogenous circRNA as an adjuvant in vaccines to elicit a more efficient immune response upon vaccination ^54^.

In all of the approaches described above, it has to be taken into consideration that side effects may arise as a result of the applied circRNA. For example, expression of the desired circRNA from an overexpression vector or translation of a protein encoded by the circRNA can significantly vary dependent on the respective cell type ^115^. In some cases, the formation of linear RNA concatemers by rolling circle transcription was also observed ^112^. Those concatemers can lead to toxic effects within the cell. The function of immune activator mentioned above can also be a drawback of circRNA if the RNA is to be applied in a context wherein an immune response is not desired.

## Conclusions

The occurrence of circRNAs in all kingdoms of life has been demonstrated, and it is beyond doubt that these abundant stable RNA species play important biological roles. The elucidation of circRNA function has included the development of methods for circRNA identification and characterization and of strategies for circRNA generation. It has become clear that circRNAs are strongly involved in diseases, although their action is enormously multifaceted. Even with all of the effort over the past decade to shed light onto this still-emerging field, the intracellular and extracellular roles of circRNAs as well as their functional role in bigger networks with other RNAs and proteins require ongoing endeavor to gain full understanding, and with that the opportunity to use circRNAs as biomarkers or therapeutic agents and targets.

## Abbreviations

ciRNA, circular RNA containing sequences from introns; circRNA, circular RNA; IRE, inverted repeats; IRES, internal ribosome entry site; MBL, muscleblind; PIE, permuted intron exon; RIG-I, retinoic acid inducible gene I; RNP, ribonucleoprotein.
